# Supplementary material for: Parkinson’s Disease Causative Mutation in Vps35 Disturbs Tetherin Trafficking to Cell Surfaces and Facilitates Virus Spread
Source: Cells. 2021 Mar 28;10(4):746. doi: 10.3390/cells10040746 (PMC8066283; doi:10.3390/cells10040746)
Supplement: Supplementary file 1 [file cells-10-00746-s001.zip › cells-1109208-Supp. Material for XML.docx]

Article

Parkinson’s Disease Causative Mutation in Vps35 Disturbs Tetherin Trafficking to Cell Surfaces and Facilitates Virus Spread

Yingzhuo Ding ^1,†^, Yan Li ^1,†^, Gaurav Chhetri ^1^, Xiaoxin Peng ^1^, Jing Wu ^1^, Zejian Wang ^1^, Bo Zhao ^1^, Wenjuan Zhao ^1^ and Xueyi Li ^1, 2,^*

| **Citation:** Ding, Y.; Li, Y.; Chhetri, G.; Peng, X.; Wu, J.; Wang, Z.; Zhao, B.; Zhao, W.; Li, X. Parkinson’s Disease Causative Mutation in Vps35 Disturbs Tetherin Trafficking to Cell Surfaces and Facilitates Virus Spread. *Cells* **2021**, *10*, x. https://doi.org/10.3390/xxxxx  Academic Editor: Wolfgang Jost, Alexander E. Kalyuzhny  Received: 28 January 2021  Accepted: 25 March 2021  Published: date  **Publisher’s Note:** MDPI stays neutral with regard to jurisdictional claims in published maps and institutional affiliations. 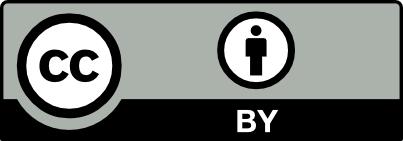 **Copyright:** © 2021 by the authors. Submitted for possible open access publication under the terms and conditions of the Creative Commons Attribution (CC BY) license (http://creativecommons.org/licenses/by/4.0/). |
| --- |
|  |

^1^ School of Pharmacy, Shanghai Jiao Tong University, Shanghai 200240, China; yingzhuoD@sjtu.edu.cn (Y.D.); 018170210001@sjtu.edu.cn (Y.L.); gvchhetri@sjtu.edu.cn (G.C.); xxpeng@sjtu.edu.cn (X.P.); wjszmwzswy@sjtu.edu.cn (J.W.); wangzejian@sjtu.edu.cn (Z.W.); bozhao@sjtu.edu.cn (B.Z.); zhaowj@sjtu.edu.cn (W.Z.)

^2^ Department of Neurology, Massachusetts General Hospital and Harvard Medical School, Charlestown, MA 02129, USA

***** Correspondence: Xueyili@sjtu.edu.cn or Xueyi.Li@mgh.harvard.edu

**†** These authors contributed equally.


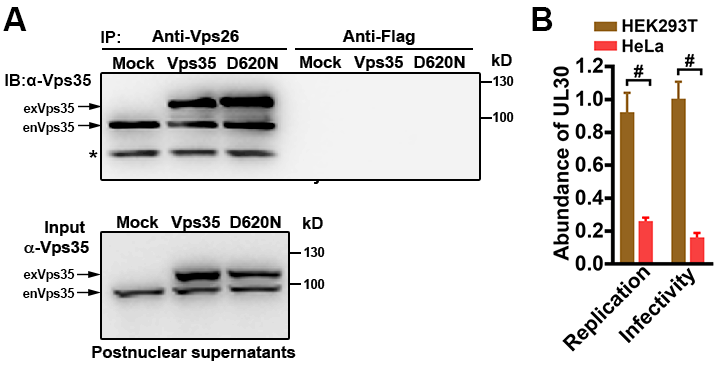


**Figure S1.** (**A**) Co-precipitation of ectopically expressed vacuolar protein sorting 35 ortholog (Vps35) with endogenous Vps26. Post-nuclear supernatants of HeLa cells transfected with plasmids expressing dsRed–Vps35 or dsRed–Vps35D620N were incubated with protein-A resins pre-coupled with antibodies for Vps26 or for FLAG. Protein complexes bound on resins were washed four times in lysis buffer, each for 15 minutes, and eluted into SDS-PAGE sample buffers for Western blot analysis with antibodies for Vps35. (**B**) HeLa cells contained and released significantly more copies of HSV-1 virions than HEK293T cells. Titers of herpes simplex virus (HSV)-1 virions were determined by relative abundance of UL30 inside initial infected cells (replication) and cells exposed to respective conditioned media for 2 hours (infectivity).


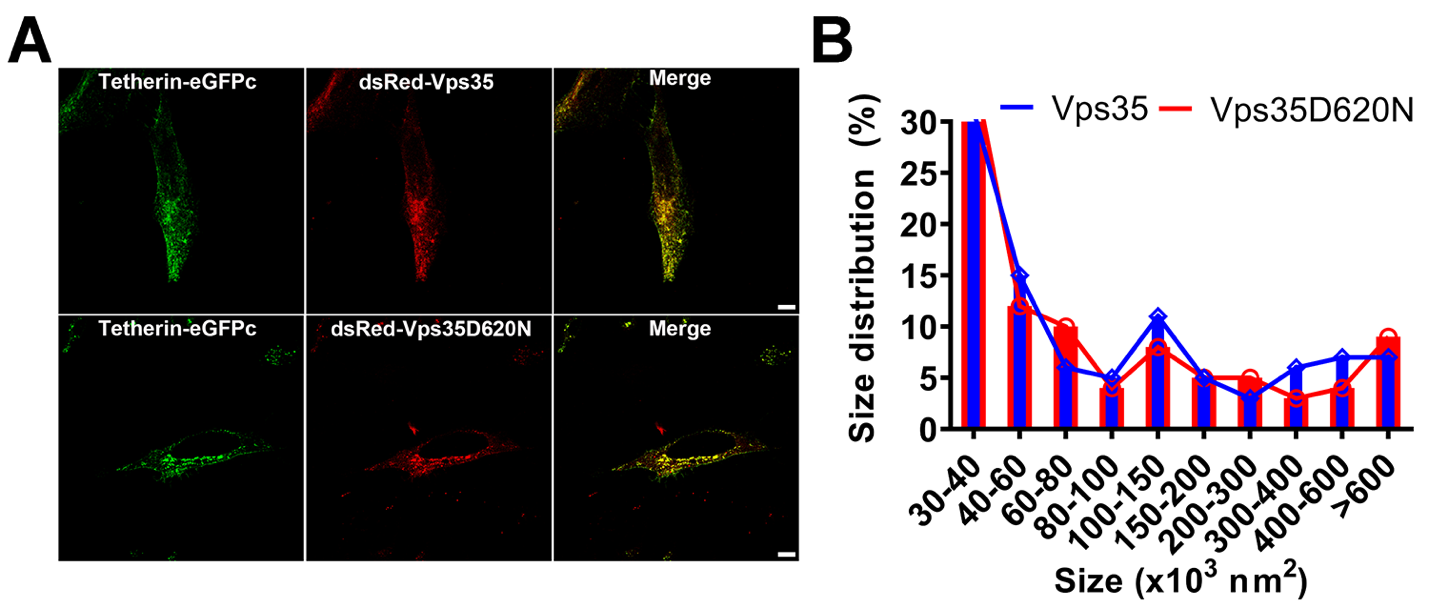


**Figure S2.** Comparison of co-localization of Tetherin–eGFPc between Vps35 wildtype and D620N mutant in fixed cells. HeLa cells on glass coverslips were transfected with plasmids expressing Tetherin–eGFPc along with plasmids expressing dsRed-Vps35 or with plasmids expressing dsRed-Vps35D620N and processed exactly the same as in Figure 6. (**A**) Confocal images. Scale bars: 10μm. (**B**) Plots show size distribution of structures co-labeled with Tetherin–GFPc and dsRed–Vps35 or with Tetherin–eGFPc and dsRed–Vps35.


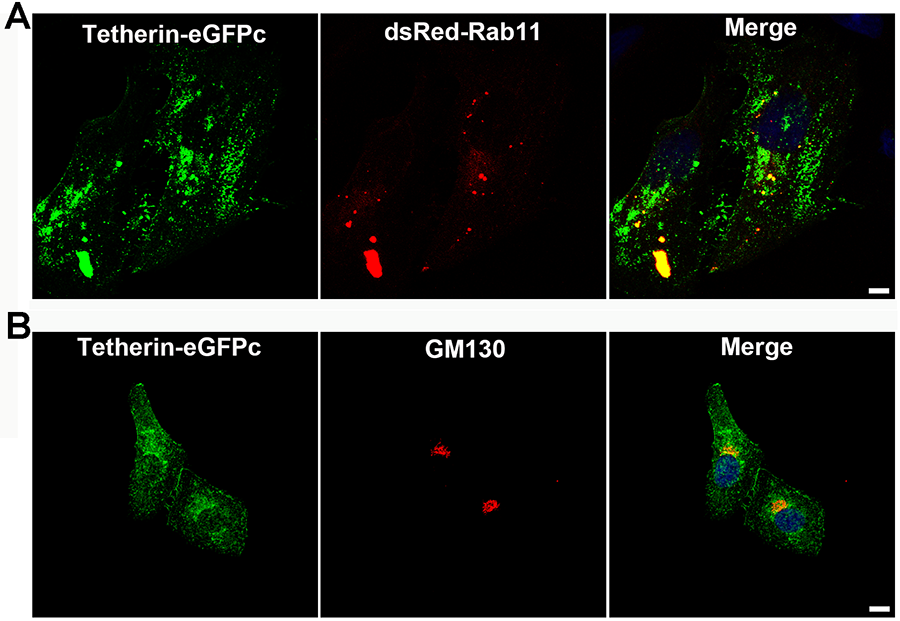


**Figure S3.** Co-localization of Tetherin–eGFPc with dsRed–Rab11 (**A**) or with GM130 (**B**). HeLa cells on glass coverslips were transfected with plasmids expressing Tetherin–eGFPc alone or together with plasmids expressing dsRed–Rab11. After being treated with cycloheximide, cells were fixed and processed for fluorescence microscopy. Cells transfected with Tetherin–eGFPc expressing plasmids alone were incubated with antibodies against GM130 followed by Cy3-conjugated secondary antibodies. Shown are confocal images. Scale bars: 10μm.

**Video S1**. Tetherin-GFPc traffics together with dsRed-Vps35

**Video S2.** Tetherin-GFPc traffics together with dsRed-Vps35D620N
